# Cardiorespiratory Comorbidity and Postoperative Complications following Esophagectomy: a European Multicenter Cohort Study

**DOI:** 10.1245/s10434-019-07478-6

**Published:** 2019-06-10

**Authors:** F. Klevebro, J. A. Elliott, A. Slaman, B. D. Vermeulen, S. Kamiya, C. Rosman, S. S. Gisbertz, P. R. Boshier, J. V. Reynolds, I. Rouvelas, G. B. Hanna, M. I. van Berge Henegouwen, S. R. Markar

**Affiliations:** 1 Department of Upper Abdominal Surgery, Centre for Digestive Diseases, Karolinska University Hospital, Stockholm, Sweden; 2 Division of Surgery, Department of Clinical Science, Intervention and Technology (CLINTEC), Karolinska Institutet, Stockholm, Sweden; 3 Department of Surgery, Trinity College Dublin, Dublin, Ireland; 4 The National Esophageal and Gastric Center, St. James’s Hospital, Dublin, Ireland; 5 Department of Surgery, Amsterdam University Medical Centers, Cancer Center Amsterdam, University of Amsterdam, Amsterdam, The Netherlands; 6 Department of Gastroenterology and Hepatology, Radboud University Medical Center, Nijmegen, The Netherlands; 7 Radboud University, Nijmegen, The Netherlands; 8 Department Surgery and Cancer, Imperial College London, London, UK

## Abstract

**Background:**

The impact of cardiorespiratory comorbidity on operative outcomes after esophagectomy remains controversial. This study investigated the effect of cardiorespiratory comorbidity on postoperative complications for patients treated for esophageal or gastroesophageal junction cancer.

**Patients and Methods:**

A European multicenter cohort study from five high-volume esophageal cancer centers including patients treated between 2010 and 2017 was conducted. The effect of cardiorespiratory comorbidity and respiratory function upon postoperative outcomes was assessed.

**Results:**

In total 1590 patients from five centers were included; 274 (17.2%) had respiratory comorbidity, and 468 (29.4%) had cardiac comorbidity. Respiratory comorbidity was associated with increased risk of overall postoperative complications, anastomotic leak, pulmonary complications, pneumonia, increased Clavien–Dindo score, and critical care and hospital length of stay. After neoadjuvant chemoradiotherapy, respiratory comorbidity was associated with increased risk of anastomotic leak [odds ratio (OR) 1.83, 95% confidence interval (CI) 1.11–3.04], pneumonia (OR 1.65, 95% CI 1.10–2.47), and any pulmonary complication (OR 1.52, 95% CI 1.04–2.22), an effect which was not observed following neoadjuvant chemotherapy or surgery alone. Cardiac comorbidity was associated with increased risk of cardiovascular and pulmonary complications, respiratory failure, and Clavien–Dindo score ≥ IIIa. Among all patients, forced expiratory volume in 1 s (FEV1)/forced vital capacity (FVC) ratio > 70% was associated with reduced risk of overall postoperative complications, cardiovascular complications, atrial fibrillation, pulmonary complications, and pneumonia.

**Conclusions:**

The results of this study suggest that cardiorespiratory comorbidity and impaired pulmonary function are associated with increased risk of postoperative complications after esophagectomy performed in high-volume European centers. Given the observed interaction with neoadjuvant approach, these data indicate a potentially modifiable index of perioperative risk.

Surgical resection remains the mainstay of curative treatment for esophageal cancer.^1^–^3^ Neoadjuvant therapy is increasingly the standard of care for patients with locally advanced disease, with neoadjuvant chemoradiotherapy in the CROSS randomized controlled trial (RCT) achieving 5-year survival of 47%, establishing a modern benchmark for treatment with curative intent.^4^ Moreover, the recent publication of the NeoFLOT data, and presentation of the FLOT 4 trial data, including patients with adenocarcinoma of the esophagogastric junction, highlighted real progress in the curative management of this cancer with pre- and postoperative chemotherapy.^5^^,^^6^

Notwithstanding this increased cure rate, esophagectomy remains an exemplar of complex major surgery associated with significant risk of major morbidity, and substantial impact on health-related quality of life.^7^^,^^8^ In this context, a number of strategies have been developed in recent years to optimize the perioperative care of patients and to minimize morbidity. These include enhanced recovery after surgery (ERAS) protocols,^9^^,^^10^ and centralization of esophageal cancer surgery to high-volume centers.^11^ In addition, the advent of total or hybrid minimally invasive approaches to esophagectomy represents a further advance, with a reduction in postoperative pulmonary infections evidenced in recent randomized trials.^12^^,^^13^

An area of controversy is whether the benefit of improved oncologic outcomes from a combination approach including neoadjuvant therapies is offset by an increase in operative complications, in particular pulmonary morbidity.^11^^,^^14^–^17^ In addition, whether preexisting respiratory and cardiac comorbidity impacts on complications for each multimodality treatment approach is unclear. Impaired pulmonary function, measured with spirometry, is a known risk factor for postoperative complications.^18^ Accordingly, the primary objective of this study is to evaluate the effect of cardiac and respiratory comorbidity on postoperative complications and mortality. The secondary objective is to evaluate the role of preoperative pulmonary function tests in identifying patients at risk of postoperative complications and mortality, and to evaluate the effects of cardiorespiratory comorbidity according to treatment approach, either surgery alone, neoadjuvant chemotherapy, or neoadjuvant chemoradiotherapy.

## Patients and Methods

### Datasets

Data were collected from 2010 to 2017 from five high-volume European esophageal cancer centers: (1) Karolinska University Hospital, Stockholm, Sweden (*n* = 400), (2) Imperial College London, UK (*n* = 137), (3) Academic Medical Center, Amsterdam, The Netherlands (*n* = 575), (4) Radboud University Medical Center, Nijmegen, The Netherlands (*n* = 79), and (5) The National Esophageal and Gastric Center, St. James’s Hospital, Dublin, Ireland (*n* = 399).

### Inclusion Criteria

All patients operated with curative intent for esophageal or esophagogastric junction (EGJ) tumors (Siewert type I and II) were included. Patients receiving surgery alone (S), neoadjuvant chemotherapy (NCS), and neoadjuvant chemoradiotherapy (NCRS) were studied. Patients receiving total minimally invasive, hybrid minimally invasive, and open esophagectomy were included.

### Exclusion Criteria

Patients found to have unresectable disease at time of surgery, those with Siewert type III EGJ tumors, or receiving definitive chemoradiotherapy followed by salvage esophagectomy were excluded.

### Clinical Staging and Follow-Up

Patients with histologically confirmed cancer of the esophagus or EGJ were staged using endoscopy and positron emission tomography (PET)–computed tomography of the thorax and abdomen, which was the standard practice at all centers. Endoscopic ultrasound was used in the staging of EGJ tumors at all centers. All centers had a standardized postoperative pathway following esophagectomy, in keeping with enhanced recovery after surgery (ERAS) principles, though there was some minor heterogeneity between centers.

Follow-up was similar between centers, with mortality identified by linking institutional datasets with national databases to ensure absolute accuracy of all-cause mortality.

### Outcomes Including Definition of Complications

The outcomes evaluated included 30-day and in-hospital complications, which were defined as: postoperative respiratory failure requiring critical care admission, postoperative atrial fibrillation requiring treatment, according to postoperative Clavien–Dindo severity classification, anastomotic leakage or conduit necrosis (endoscopically or radiographically verified), reoperation for any cause, pneumonia defined by the individual investigator when at least one of the following criteria were fulfilled: new and persistent or progressive and persistent radiographic infiltrate, and at least one of: fever (> 38.0 °C or > 100.4 °F), leukopenia (≤ 4000 WBC/mm^3^), or leukocytosis (> 12,000 WBC/mm^3^), for adults > 70 years old, altered mental status with no other recognized cause, and at least two of the following: new onset of purulent sputum or change in character of sputum, or increased respiratory secretions, or increased suctioning requirements, new-onset or worsening cough, or dyspnea, or tachypnea, rales or bronchial breath sounds, worsening gas exchange [e.g., O_2_ desaturations (e.g., PaO_2_/FiO_2_ < 240)], increased oxygen requirements, or increased ventilator demand.^19^ In-hospital, 30-day, and 90-day postoperative all-cause mortality were compiled with complete follow-up. Length of intensive care unit and hospital stay were measured with complete follow-up.

### Exposure

The exposure of the study was cardiac or respiratory disease recorded at baseline. Respiratory disease was defined as chronic pulmonary disease with impaired lung function, including mild, moderate, or severe chronic obstructive lung disease, pulmonary fibrosis, severe asthma, or other chronic pulmonary disease assessed by the surgeon at the initial consultation for esophageal cancer. Cardiac disease was defined as chronic heart disease with impaired cardiac function, including previous myocardial infarction, congestive heart failure, or other chronic cardiac disease assessed by the physician assessing the patient for esophagectomy. Classification of comorbidities was performed by the researcher based on the available clinical data at each site (authors F. Klevebro, J. A. Elliott, A. Slaman, B. D. Vermeulen, P. R. Boshier, and S. R. Markar).

### Covariates

Sex, age, weight, smoking status (smoker/nonsmoker/ex-smoker), American Society of Anesthesiologists (ASA) grade, Eastern Cooperative Oncology Group (ECOG) performance status, and comorbidities including hypertension and diabetes were collected. Preoperative results on spirometry tests [FVC, FEV1, Tiffeneau–Pinelli index (FEV1/FVC), bicycle or treadmill ergometry results (measured in W/kg), and left ventricular ejection fraction (LVEF) measured by echocardiogram (%)] were compiled when available. Patients were stratified by FEV1/FVC ratio > 70%^20^ and LVEF > 55%.^21^ Operative data including surgical technique were gathered.

### Statistical Analysis

The following confounders with categorizations were used in the adjusted model for the main analysis: age (continuous, years), gender [categorical: male (reference)/female], histology [categorical: adenocarcinoma (reference)/squamous cell carcinoma], clinical T-stage (categorical: 0–4), clinical N-stage (categorical: 0–3), surgical technique [transthoracic esophagectomy according to Ivor Lewis (reference), three-field esophagectomy according to McKeown, or transhiatal esophagectomy], and surgical approach [open (reference), hybrid minimally invasive, or total minimally invasive esophagectomy].

For categorical outcomes, logistic regression models were used to calculate odds ratios (OR) with 95% confidence intervals (CI). All above-mentioned regression models were adjusted for predefined confounders.

Approval was granted from the regional research ethics committee of each participating center.

## Results

### Comparison of Baseline Patient Demographics and Pretreatment Tumor Stage

#### Preoperative Patient Demographics

Significant underlying differences in patient age, sex, and ASA score were observed when comparing patients with and without cardiorespiratory comorbidity (Table 1).

**Table 1:** Unadjusted comparison of patient and treatment factors stratified by preoperative cardiorespiratory comorbidity status

*N* (%)	No cardiorespiratory comorbidity (*N* = 982)	Respiratory comorbidity (*N* = 274)	Cardiac comorbidity (*N* = 468)	Cardiorespiratory comorbidity (*N* = 608)	*P* value*
Age (median, IQR), years	63 (56–70)	65 (60–71)	67 (62–72)	67 (61–71)	< 0.001
Sex					0.001
Male	739 (75.3)	219 (79.9)	392 (83.8)	500 (82.2)	
Female	243 (24.8)	55 (20.1)	76 (16.2)	108 (17.8)	
ASA score					< 0.001
1	7 (0.7)	26 (9.5)	24 (5.1)	46 (7.6)	
2	345 (35.1)	160 (58.4)	270 (57.7)	356 (58.6)	
3	515 (52.4)	87 (31.8)	171 (36.5)	203 (33.4)	
4	113 (11.5)	1 (0.4)	3 (0.6)	3 (0.5)	
ECOG score (*n* = 1264)					0.094
0	582 (70.7)	168 (74.0)	227 (69.4)	319 (72.3)	
1	229 (27.8)	51 (22.5)	88 (26.9)	108 (24.5)	
2	12 (1.5)	7 (3.1)	11 (3.4)	13 (3.0)	
3	0 (0)	1 (0.4)	1 (0.3)	1 (0.2)	
Smoking (*n* = 850)					0.086
Never	158 (34.4)	65 (28.4)	76 (27.2)	107 (27.4)	
Former	211 (46.0)	104 (45.4)	157 (56.3)	200 (51.2)	
Currently	90 (19.6)	60 (26.2)	46 (16.5)	84 (21.5)	
FVC L, median (IQR)	4.1 (3.2–4.7)	3.7 (3.1–4.4)	3.8 (3.4–4.3)	3.7 (3.2–4.3)	0.008
FEV1 L, median (IQR)	2.9 (2.4–3.5)	2.4 (1.9–3.1)	2.8 (2.1–3.1)	2.6 (2.1–3.1)	0.020
FEV1/FVC median % (IQR) (*n* = 850)	83.3 (74.3–97.0)	73.3 (63.2–82.6)	88.0 (75.9–100)	84 (71.7–98.0)	0.747
Bicycle test (W) (*n* = 34)	No observations	129 (114–156)	131 (118–157)	131 (117–160)	
Left ventricular ejection fraction (%) (*n* = 234)	59.5 (55.0–63.0)	59 (55.0–60.0)	55 (50.0–60.0)	56.5 (53.0–60.0)	0.056
cT					0.127
1	111 (11.3)	44 (16.1)	62 (13.3)	81 (13.3)	
2	173 (17.6)	42 (15.3)	75 (16.0)	103 (16.9)	
3	614 (62.5)	168 (61.3)	308 (65.8)	392 (64.5)	
4	48 (4.9)	12 (4.4)	10 (2.1)	18 (3.0)	
X	36 (3.7)	8 (2.9)	13 (2.8)	14 (2.3)	
cN					0.057
0	438 (44.6)	125 (45.6)	182 (38.9)	244 (40.1)	
1	394 (40.1)	121 (44.2)	224 (47.9)	286 (47.0)	
2	133 (13.5)	22 (8.0)	57 (12.2)	69 (11.4)	
3	17 (1.7)	6 (2.2)	5 (1.1)	9 (1.5)	
Tumor type					0.125
Adenocarcinoma	747 (76.1)	209 (76.3)	367 (78.2)	475 (78.1)	
SCC	207 (21.1)	62 (22.6)	94 (20.1)	125 (20.6)	
Other	28 (2.9)	3 (1.1)	7 (1.5)	8 (1.3)	
Tumor location					< 0.001
Upper/middle	142 (14.5)	35 (12.8)	48 (10.3)	66 (10.9)	
Distal	326 (33.2)	88 (32.1)	253 (54.1)	297 (48.9)	
EGJ	514 (52.3)	151 (55.1)	167 (35.7)	245 (40.3)	
Surgical approach					0.005
Open	527 (53.7)	194 (70.8)	223 (47.7)	338 (55.6)	
HMIO	36 (3.7)	5 (1.8)	5 (1.1)	6 (1.0)	
TMIO	419 (42.7)	75 (27.4)	240 (51.3)	264 (43.4)	
Surgical technique					< 0.001
Ivor Lewis	564 (57.5)	139 (51.1)	228 (48.8)	293 (48.4)	
McKeown	305 (31.1)	62 (22.8)	140 (30.0)	179 (29.5)	
Transhiatal	112 (11.4)	71 (26.1)	99 (21.2)	134 (22.1)	
Neoadjuvant treatment					0.095
None	232 (23.6)	81 (29.6)	105 (22.4)	137 (22.5)	
Chemotherapy	182 (18.5)	54 (19.7)	54 (11.5)	90 (14.8)	
Chemoradiotherapy	568 (57.8)	139 (50.7)	309 (66.0)	381 (62.7)	

*IQR* interquartile range, *HMIO* hybrid minimally invasive esophagectomy, *TMIO* totally minimally invasive esophagectomy, *SCC* squamous cell carcinoma

*Comparing patients with and without preoperative cardiopulmonary disease

#### Pretreatment Tumor Characteristics and Surgical Approach

There were no statistically significant differences in tumor stage, tumor type, or use of neoadjuvant treatment between the groups, however proximal tumor location was less common in the cardiorespiratory comorbidity group (Table 1). Surgical technique did show variation between the groups, with the cardiorespiratory comorbidity group having a higher proportion of transhiatal resections (22.1% vs. 11.4%, *P* < 0.001, Table 1).

### Comparison of Postoperative Outcomes

#### Respiratory Comorbidity and Postoperative Outcomes

Preoperative respiratory comorbidity was associated with an increase in postoperative complications (70.8% vs. 64.4%), length of intensive care unit stay (median 1 day vs. 0 days), and length of hospital stay (median 18 days vs. 14 days). Postoperative Clavien–Dindo complication scores were significantly increased among patients with baseline respiratory comorbidity (*P* = 0.037, Table 2). Multivariable adjusted analyses showed an increased risk of anastomotic leak (OR 1.64, 95% CI 1.11–2.41) and pneumonia (OR 1.39, 95% CI 1.04–1.85, Table 3) among patients with baseline respiratory comorbidity.

**Table 2:** Comparison of postoperative outcomes stratified by preoperative cardiorespiratory comorbidity status

*N* (%)	No cardiorespiratory comorbidity (*N* = 982)	Respiratory comorbidity (*N* = 274)	Cardiac comorbidity (*N* = 468)	Cardiorespiratory comorbidity (*N* = 608)	*P* value*
In-hospital mortality	28 (2.9)	12 (4.4)	24 (5.1)**	26 (4.3)	0.128
Postoperative complication	632 (64.4)	194 (70.8)**	305 (65.2)	408 (67.1)	0.263
Length of intensive care unit stay, median days (IQR)	0 (0–2)	1 (0–5)**	1 (0–5)	1 (0–5)**	< 0.001
Length of hospital stay, median days (IQR)	14 (10–22)	18 (12–30)**	15 (9–27)	15 (10–27)	0.057
Reoperation	48 (7.1)	20 (10.7)	47 (11.8)**	59 (11.4)**	0.010
Pneumonia	255 (26.0)	98 (35.8)**	136 (29.1)	180 (29.6)	0.114
Anastomotic leak	111 (11.3)	42 (15.3)	73 (15.6)**	86 (14.1)	0.095
Conduit necrosis	6 (0.9)	2 (2.7)	19 (4.7)**	19 (3.7)**	0.001
Cardiovascular complication	174 (17.7)	57 (20.9)	129 (27.6)**	153 (25.2)**	< 0.001
Atrial fibrillation	109 (11.1)	32 (11.7)	49 (10.5)	67 (11.0)	0.961
Respiratory failure	95 (9.7)	41 (15.0)**	66 (14.1)**	81 (13.3)**	0.024
Pulmonary complication	343 (34.9)	122 (44.5)**	200 (42.7)**	254 (41.8)**	0.006
Clavien–Dindo score	–	*P* = 0.037**	*P* < 0.001**		*P* < 0.001
Grade I	216 (28.0)	64 (26.7)	75 (26.9)	105 (22.8)	
Grade II	231 (29.9)	68 (28.3)	76 (22.2)	114 (24.7)	
Grade IIIa	143 (18.5)	27 (11.3)	46 (13.5)	64 (13.9)	
Grade IIIb	56 (7.3)	22 (9.2)	19 (5.6)	30 (6.5)	
Grade IVa	93 (12.1)	45 (18.8)	84 (24.6)	100 (21.7)	
Grade IVb	25 (3.2)	13 (5.4)	26 (7.6)	32 (6.9)	
Grade V	8 (1.0)	1 (0.42)	16 (4.7)	16 (3.5)	
Clavien–Dindo score, median (IQR)	II (I–IIIa)	II (I–IIIb)	IIIa (II–IVa)	IIIa (II–IVa)	< 0.001

*Comparing patients with and without preoperative cardiopulmonary disease

**P < 0.05 comparing patients with and without preoperative cardiopulmonary disease

**Table 3:** Multivariable adjusted analyses of the association between preoperative cardiorespiratory comorbidity and pulmonary function with postoperative complications and Clavien–Dindo scores

Odds ratio (95% CI)^a^	In-hospital mortality	Postoperative complication	Anastomotic leak	Cardiovascular complication	Atrial fibrillation	Pulmonary complication	Pneumonia	Respiratory failure	Clavien–Dindo grade ≥ IIIa
Respiratory comorbidity^b^	1.36 (0.69–2.67)	1.21 (0.91–1.63)	1.64 (1.11–2.41)	0.88 (0.63–1.23)	0.74 (0.48–1.13)	1.26 (0.96–1.66)	1.39 (1.04–1.85)	1.43 (0.97–2.09)	0.95 (0.71–1.27)
Cardiac comorbidity^c^	1.54 (0.88–2.72)	0.95 (0.75–1.21)	1.25 (0.90–1.72)	1.63 (1.25–2.13)	0.88 (0.60–1.29)	1.44 (1.14–1.82)	1.19 (0.92–1.53)	1.49 (1.06–2.09)	1.73 (1.34–2.25)

^a^Adjusted for age (continuous), sex (male or female), cT stage (0,1,2,3,4), cN stage (0,1,2,3), tumor histology (adenocarcinoma, SCC, other), surgical approach (open, HMIO, TMIO), and surgical technique (Ivor Lewis, McKeown, transhiatal)

^b^Comparing patients with and without preoperative respiratory disease

^c^Comparing patients with and without preoperative cardiac disease

#### Cardiac Comorbidity

Patients with preoperative cardiac comorbidity had significantly increased risk of in-hospital mortality (5.1% vs. 2.7%), anastomotic leak (15.6 vs. 11.3%), conduit necrosis (4.7% vs. 0.9%), cardiovascular complications (27.6% vs. 17.7%), respiratory failure (14.1% vs. 9.7%), reoperation for any cause (11.8% vs. 7.1%), and any pulmonary complication (42.7% vs. 34.9%). Postoperative Clavien–Dindo complication scores were also increased with cardiac comorbidity (*P* < 0.001, Table 2). Multivariable adjusted analyses showed an increased risk of cardiovascular complications (OR 1.63, 95% CI 1.25–2.13), pulmonary complications (OR 1.44, 95% CI 1.14–1.82), respiratory failure (OR 1.49, 95% CI 1.06–2.09), and Clavien–Dindo score ≥ IIIa (OR 1.73, 95% CI 1.34–2.25, Table 3) among patients with preoperative cardiac comorbidity.

#### Cardiorespiratory Comorbidity among Postoperative Complications According to Neoadjuvant Protocol

In a multivariable adjusted analysis, respiratory comorbidity was associated with increased risk of anastomotic leak (OR 1.83, 95% CI 1.11–3.04), pneumonia (OR 1.65, 95% CI 1.10–2.47), and any pulmonary complication (OR 1.52, 95% CI 1.04–2.22) after neoadjuvant chemoradiotherapy but not after neoadjuvant chemotherapy or surgery alone (Table 4).

**Table 4:** Multivariable analyses to evaluate the effect of cardiac and respiratory comorbidity on postoperative outcomes stratified by neoadjuvant treatment

Odds ratio (95% CI)^a^	In-hospital mortality	Postoperative complication	Anastomotic leak	Cardiovascular complication	Atrial fibrillation OR (95% CI)	Pulmonary complication	Pneumonia	Respiratory failure	Clavien–Dindo grade ≥ IIIa
*Surgery alone*									
Respiratory comorbidity	1.84 (0.60–5.95)	0.84 (0.48–1.45)	1.45 (0.70–2.99)	0.74 (0.39–1.40)	0.74 (0.35–1.56)	1.00 (0.59–1.67)	1.25 (0.72–2.16)	0.83 (0.39–1.79)	0.95 (0.56–1.63)
Cardiac comorbidity	0.95 (0.30–2.97)	1.00 (0.60–1.69)	0.89 (0.45–1.79)	1.75 (1.01–3.04)	1.07 (0.55–2.10)	1.69 (1.05–2.71)	1.41 (0.85–2.35)	1.53 (0.79–2.95)	1.80 (1.07–3.04)
*Neoadjuvant chemotherapy*									
Respiratory comorbidity	2.03 (0.29–14.40)	1.60 (0.73–3.37)	1.46 (0.43–4.94)	0.90 (0.42–1.90)	0.84 (0.39–1.84)	0.96 (0.51–1.81)	0.91 (0.48–1.74)	1.87 (0.79–4–43)	0.90 (0.47–1.75)
Cardiac comorbidity	12.62 (1.30–117.80)	1.49 (0.70–3.15)	1.51 (0.44–5.10)	2.00 (1.01–3.96)	1.55 (0.76–3.18)	1.57 (0.83–2.98)	1.62 (0.85–3.08)	2.35 (0.98–5–65)	1.96 (0.99–3.88)
*Neoadjuvant chemoradiotherapy*									
Respiratory comorbidity	1.04 (0.38–2.82)	1.28 (0.85–1.90)	1.83 (1.11–3.04)	0.97 (0.61–1.55)	0.62 (0.29–1.32)	1.52 (1.04–2.22)	1.65 (1.10–2.47)	1.60 (0.94–2.74)	0.91 (0.60–1.37)
Cardiac comorbidity	1.56 (0.75–3.27)	0.92 (0.68–1.23)	1.34 (0.90–2.00)	1.48 (1.04–2.10)	0.53 (0.27–1.03)	1.34 (0.99–1.81)	1.06 (0.75–1.49)	1.41 (0.89–2.22)	1.73 (1.23–2.44)

^a^Adjusted for age (continuous), sex (male or female), cT stage (0,1,2,3,4), cN stage (0,1,2,3), tumor histology (adenocarcinoma, SCC, other), surgical approach (open, HMIO, TMIO), and surgical technique (Ivor Lewis, McKeown, transhiatal)

Cardiac comorbidity was associated with increased risk of cardiovascular complications, pulmonary complications, and Clavien–Dindo score ≥ IIIa regardless of neoadjuvant treatment. In the neoadjuvant chemotherapy group, cardiac comorbidity was associated with increased risk of in-hospital mortality (OR 12.62, 95% CI 1.30–117.80, Table 4).

### Preoperative Respiratory and Cardiac Investigations and Postoperative Outcomes

Of 1590 patients, 608 (38.2%) had a cardiorespiratory comorbidity, of whom 407 (66.9%) had documented preoperative pulmonary function testing and 106 (17.4%) had a preoperative echocardiogram. Ergometry results were only available for 34 (5.6%) patients and were not included in the analyses. Unadjusted analyses showed that FEV1/FVC ratio > 70% was associated with reduced overall postoperative complications, cardiovascular complications, atrial fibrillation, pulmonary complications, and pneumonia. Adjusted analyses showed that FEV1/FVC ratio > 70% was associated with reduced risk of overall postoperative complications (OR 0.57, 95% CI 0.37–0.89) and atrial fibrillation (OR 0.46, 95% CI 0.28–0.75, Table 5). LVEF > 55% was associated with decreased risk of anastomotic leak (adjusted OR 0.34, 95% CI 0.12–0.95), but was otherwise unrelated to postoperative outcomes.

**Table 5:** Univariable and multivariable analyses to evaluate the effect of preoperative respiratory function on postoperative outcomes

Odds ratio (95% CI)	In-hospital mortality	Postoperative complication	Anastomotic leak	Cardiovascular complication	Atrial fibrillation	Pulmonary complication	Pneumonia	Respiratory failure	Clavien–Dindo grade ≥ IIIa
*All patients unadjusted*									
FEV1/FVC ratio									
≤ 70.0% (ref)	–	–	–	–	–	–	–	–	–
> 70.0% LVEF	0.91 (0.30–2.75)	0.44 (0.29–0.66)	1.28 (0.69–2.38)	0.57 (0.39–0.84)	0.22 (0.14–0.34)	0.61(0.43–0.86)	0.48 (0.33–0.70)	0.59 (0.35–1.01)	1.41 (0.95–2.08)
≤ 55.0% (ref)	–	–	–	–	–	–	–	–	–
> 55.0%	0.96 (0.21–4.39)	0.58 (0.31–1.09)	0.33 (0.12–0.91)	0.98 (0.56–1.72)	1.04 (0.59–1.85)	0.95 (0.56–1.59)	0.91 (0.53–1.56)	0.54 (0.24–1.20)	0.90 (0.50–1.62)
*All patients adjusted* ^*a*^									
FEV1/FVC ratio									
≤ 70.0% (ref)	–	–	–	–	–	–	–	–	–
> 70.0% LVEF	1.13 (0.34–3.73)	0.57 (0.37–0.89)	0.79 (0.40–1.56)	0.73 (0.48–1.11)	0.46 (0.28–0.75)	0.76 (0.52–1.11)	0.72 (0.48–1.07)	0.76 (0.43–1.34)	1.24 (0.82–1.89)
≤ 55.0% (ref)	–	–	–	–	–	–	–	–	–
> 55.0%	0.74 (0.15–3.71)	0.55 (0.28–1.07)	0.34 (0.12–0.95)	1.04 (0.57–1.89)	1.11 (0.61–2.03)	0.97 (0.57–1.65)	0.91 (0.52–1.60)	0.56 (0.24–1.28)	0.94 (0.51–1.72)

^a^Adjusted for age (continuous), sex (male or female), cT stage (0,1,2,3,4), cN stage (0,1,2,3), tumor histology (adenocarcinoma, SCC, other), surgical approach (open, HMIO, TMIO), and surgical technique (Ivor Lewis, McKeown, transhiatal)

FEV1/FVC ratio *N* = 849, LVEF *N* = 234

## Discussion

This large multicenter cohort study from high-volume European academic centers highlights several important points. First, cardiac or respiratory comorbidity substantially increased the risk and severity of postoperative complications, among patients deemed fit ab initio for surgery. Second, impaired pulmonary function based on spirometry reliably predicted pulmonary and cardiovascular complications, atrial fibrillation, pneumonia, and overall postoperative complications. Third, neoadjuvant chemoradiotherapy was associated with increased risk of anastomotic leak and pulmonary complications among patients with respiratory comorbidity, while neoadjuvant chemotherapy or surgery alone was not.

Although risk assessment as part of modern management and ERAS programs for esophageal surgery has become increasingly standardized, accurate prediction of risk remains a significant clinical challenge.^11^^,^^17^ Age alone has not been associated with increased postoperative risk in most studies.^22^^,^^23^ Higher ASA score and decreased performance status are however established risk factors, and this was confirmed in the present study (data not shown).^11^ Preoperative assessment using spirometry, or bicycle or treadmill ergometry is commonly used to evaluate the patient’s fitness to undergo esophagectomy.^18^^,^^24^ Interestingly, however, even among academic centers, approximately only two-thirds of patients with preoperative cardiac or respiratory disease underwent such evaluation in this time period. For those measures, FEV1/FVC ratio > 70% was independently associated with a decreased risk of overall postoperative complications, cardiovascular complications, atrial fibrillation, pulmonary complications, and pneumonia. It seems prudent that all patients undergoing treatment with curative intent for esophageal cancer should undergo pulmonary physiology studies, ideally before and after neoadjuvant therapy. Cardiopulmonary exercise testing (CPET) applies analysis of breath-by-breath expired gas data to evaluate cardiorespiratory capacity. This test might provide a more accurate and useful estimate of cardiorespiratory function and risks associated with esophageal cancer treatment.^25^^,^^26^ The 6-min walk test is another test of cardiorespiratory capacity and physical function, which have been shown to predict perioperative risk in thoracic surgery.^27^^,^^28^ The role of these tests in esophageal cancer treatment should be evaluated in prospective studies and in trials of prehabilitation.

Moreover, based on the present data, studies targeting preoperative optimization through prehabilitation programs, including exercise interventions and smoking cessation, to improve pulmonary function, have considerable therapeutic rationale. A randomized trial that assessed the effect of preoperative inspiratory muscle training showed improved inspiratory muscle strength but no difference in lung function parameters or pulmonary complications.^29^ A recent randomized controlled trial showed that prehabilitation with an exercise program and nutritional support significantly increased patients’ results on the 6-min walk test.^30^ Preoperative optimization with exercise, nutritional support, and the interaction with cardiorespiratory comorbidity represent a highly pertinent future research area.

In this study, cardiac comorbidity was associated with increased in-hospital mortality after neoadjuvant chemotherapy and esophagectomy. New-onset atrial fibrillation may be the most common specific complication after esophagectomy, affecting approximately 20% of patients.^31^ Preoperative treatment with 5-fluorouracil (5-FU) and platins is known to cause cardiac sensitization.^32^ It is interesting that this was not seen in the NCRS group, a fact that might be explained by the differences in the chemotherapy regimens which are used concomitant with radiotherapy. Furthermore, cytotoxic agents may impair myocellular proliferation by disrupting the mammalian target of rapamycin kinase pathway,^33^ while cisplatin also negatively impacts muscle function through a number of mechanisms including impaired Akt phosphorylation, leading to sustained activation of the degradative proteasome and autophagy systems, and altered nuclear factor kappa-light-chain-enhancer of activated B cells (NF-kB) signaling.^34^^,^^35^ Together these mechanisms may contribute to myocardial dysfunction among patients treated with preoperative chemotherapy.^24^^,^^32^ There was an increased risk for anastomotic leak, pulmonary complications, and pneumonia for patients with respiratory comorbidity after NCRS but not after NCS. This could be an effect of the added radiotherapy, which has been shown in previous studies to be associated with increased risk for radiation pneumonitis, reduced physical endurance, pulmonary diffusion capacity, and heart function, and increased risk for severe postoperative complications after esophagectomy compared with NCS.^14^^,^^17^^,^^36^–^42^ Modern neoadjuvant chemoradiotherapy regimens for esophageal cancer appear to result in specific reductions in pulmonary diffusion capacity, particularly among older adults and those with a history of smoking or preexisting lung disease, which may limit such patients’ ability to tolerate pulmonary morbidity, increasing the risk of postoperative respiratory failure.^42^ Detailed analysis of postoperative outcomes is embedded in ongoing randomized controlled trials comparing NCRS with NCS for patients with esophageal adenocarcinoma,^43^^,^^44^ and these secondary outcome measures will be of critical importance, particularly if there is oncologic equivalence.

The limitations of this study include the retrospective design and the difficulties in defining and measuring postoperative outcomes, however it must be noted that coding of complications was standardized for this study in accordance with international consensus recommendations.^45^ The included patients have been evaluated according to predefined variables by a devoted researcher at each participating center to increase the internal validity of the study. Pretreatment cardiac function tests were not available in the majority of patients, and cardiorespiratory comorbidity was assessed clinically. It is possible that cardiorespiratory comorbidity status may have been misclassified in some patients. Pulmonary function tests (PFTs) were however available in 67% of the patients. This could lead to understatement of the positive associations in the study, and prevents stratified analyses by type and function of the cardiorespiratory comorbidities. The small number of patients receiving preoperative cardiac investigations prevented robust investigation of the link between objective measurements of cardiac function and postoperative outcome. Furthermore, diffusion capacity for carbon monoxide (DLCO) was often not available, which prevented meaningful analyses of the association with postoperative complications which has been demonstrated in previous research.^46^ Strengths of the study include the standardized classification of complications, the large sample size, and the multicenter design conferring external validity to the study within high-volume European centers.

In conclusion, this study shows that cardiorespiratory comorbidity is a clear risk factor for postoperative complications after esophagectomy. Impaired preoperative lung function is associated with increased risk of postoperative complications, particularly following neoadjuvant chemoradiotherapy. Careful clinical assessment, with thorough investigation of cardiorespiratory function, is required to facilitate treatment planning for patients considered for multimodal therapy for locally advanced esophageal cancer.
